# DOM hydrophilic components of organic fertilizers increased the soil nitrogen retention capacity and succession of the microbial community

**DOI:** 10.3389/fmicb.2023.1320302

**Published:** 2023-12-06

**Authors:** Yuyun Wang, Yingtong Ge, Yaqin Deng, Xiang Xu, Yong Zhang, Lan Li, Zhi Xu

**Affiliations:** ^1^College of Resources and Environmental Science, Yunnan Agricultural University, Kunming, China; ^2^Key Laboratory of Vegetable Biology of Yunnan Province, Yunnan Agricultural University, Kunming, China

**Keywords:** organic fertilizer DOM, nitrogen retention capacity, microbiological community succession, characteristics of nitrogen transformation, soil N

## Abstract

**Introduction:**

Application of organic fertilizers affects soil properties and microbial communities, which in turn alters soil N transformation processes. Unfortunately, it is not clear how the difference in the character of the organic fertilizer DOM affects the soil nitrogen retention capacity and its microbial processes.

**Methods:**

According to the principle of equal nutrients, the treatments of chemical fertilizer alone (treatment CF), chemical fertilizer with organic fertilizer DOM hydrophilic components (treatment H), and chemical fertilizer with organic fertilizer DOM hydrophobic components (treatment P) were set up, where the characteristics of soil nitrogen transformation and changes in microbial community structure were studied with soil culture conditions for 24 days.

**Results:**

It was discovered that the addition of organic fertilizer DOM components (H and P) slowed nitrification rate and increased protease activity resulting in a higher NH_4_^+^-N content compared to the CF treatment. The DOM addition (H and P) increased the microbial biomass nitrogen (MBN) levels in the soil and increased the soil nitrogen pool capacity.

**Conclusions:**

Moreover, the carbon use efficiency of the hydrophilic components is higher than that of the hydrophobic components, resulting in its further increase in nitrogen reservoir capacity and higher nitrogen retention capacity. Network analysis showed that the addition of organic fertilizer DOM hydrophilic components increased network complexity and synergy between microorganisms. In combination with random forest analysis, it was shown that *Sphingomonas* and *Massilia* were key species influencing soil nitrogen retention capacity and nitrogen availability characteristics.

## 1 Introduction

Nitrogen (N) is considered to be one of the major factors affecting crop growth (Abbruzzini et al., 2019). China is the world’s largest consumer of fertilizer (Cui et al., 2021), and ranks in the highest position for N application in the world (Dong et al., 2020). However, the unbalanced relationship between intensive application of chemical nitrogen fertilizer and crop nitrogen requirement leads to low efficiency of N fertilizer. Applying organic fertilizer (C) has a dramatic impact on the soil nitrogen cycle, as it regulates the soil carbon and nitrogen balance, stimulates microbial activity, enhances microbial fixation of soil nitrogen and aligns nitrogen supply with crop requirements (Singh et al., 2007). A range of interconnected N transformation processes are primarily driven by soil microorganisms, including N hydrolysis and fixation, nitrification and denitrification (Kuypers et al., 2018). However, there is no clear description of the effects of organic fertilizer application on soil microorganisms that mediating nitrogen cycle, and how these changes affect soil nitrogen supply characteristics.

Carbon inputs can potentially stimulate microbial activity and N cycle by increasing the abundance of N-fixing, nitrifying and denitrifying microbiota (Chen et al., 2022). A study of C and N cycles under the influence of long-term organic fertilizer application found that manure application altered C fixation and degradation processes and promoted nitrate reduction (Hu et al., 2022). The application of organic fertilizer increased the content of organic N in soil, and thus changed the relative ratio of organic N to inorganic N in the soil (Ma et al., 2018), affecting the C:N stoichiometric ratio of soil microorganisms and their energy source. Promoting microbial reproduction and the greater species diversity can also stimulate a wide range of enzyme-mediated microbial processes (Farrell et al., 2014). However, the relationship between microbial and nitrogen cycle changes after organic fertilizer application has been controversial. Studies have shown that organic fertilizer application changed microbial communities, promoted organic matter decomposition, ureolysis and denitrification, reduced nitrification and N retention capacity, decreased nitrate N content in the soil but had no effect on ammonium N content (Huang et al., 2022). However, another study found that, compared with applying chemical fertilizer alone, the application of organic fertilizer increased microbial biomass and significantly reduced soil ammonium nitrogen content while slowing nitrification, thus promoting soil nitrogen storage (Wang J. et al., 2023). Therefore, how soil nitrogen retention capacity changes after organic fertilizer application needs to be further clarified.

Soil dissolved organic matter (DOM) provides nutrients and living environment for soil microorganisms, and influences the microbial processes of soil N transformation (Rees and Parker, 2005). Removal of DOM significantly reduced the cumulative mineralization of N in soil (Shang et al., 2015), while adding DOM increased the content of inorganic N (NH_4_^+^-N, NO_3_^–^-N) in soil (Qiu et al., 2015). This suggested that soil DOM could affect soil nitrogen supply characteristics. Organic fertilizer is produced through the composting process, and the DOM produced in the composting process is one of the main sources of soil DOM. In the composting process, DOM content increased in the early stage and then decreased gradually (Gong et al., 2021). With the composting progresses, the amount of structurally complex macromolecules and humic substances in DOM increases (Yu et al., 2011). Based on this pattern, it can be predicted that the absolute content of DOM decreases during the composting process, and the hydrophilic components decreases while the hydrophobic components increases. However, there is a clear difference in the bioavailability of the hydrophilic and hydrophobic components of organic fertilizer DOM (Zheng et al., 2021), with hydrophilic components are easier to utilize because of their higher carbon utilization efficiency by microorganisms (Zhao et al., 2008; Zheng et al., 2021). Nevertheless, the effects of organic fertilizer-derived DOM with different properties on soil nitrogen retention capacity and its microbial processes are still unclear.

Dissolved organic matter from the organic fertilizer production process (composting) is a direct source of soil DOM and is important in explaining changes in soil nitrogen cycle after organic fertilizer application. Based on this, the aim of this study was to investigate the effects of organic fertilizer DOM on the characteristics of soil nitrogen transformation and its microbial processes. Considered the bioavailability of compositions of organic fertilizer DOM are different, this study first used resin XAD-8 to separate hydrophilic and hydrophobic components of organic fertilizer DOM, and then investigated the changes in nitrogen transformation characteristics and their microbial processes after adding hydrophilic or hydrophobic components. The hypotheses of this study were that (1) compared with the single application of chemical fertilizer, adding organic fertilizer DOM compositions would increase the microbial requirements for N, which would enhance the microbial assimilation of N and allow the nitrogen to be retained in the microbes; and hydrophilic component would have stronger soil nitrogen retention capacity as it has higher bioavailability of carbon than that of hydrophobic component. (2) The hydrophilic and hydrophobic components of DOM provide different living substrates for N-transforming microorganisms, resulting in differences in the succession of microbial communities, and then changes the process of soil nitrogen cycling mediated by microorganisms. The results of this study can improve the understanding of the microbial processes of soil nitrogen transformation characteristics altered by the application of organic fertilizer.

## 2 Materials and methods

### 2.1 Sample collection and preparation

The soil samples were obtained from 0 to 20 cm soil layer of local dryland in Yunnan, China (102.74°E, 25.12°N). This region has a typical subtropical plateau monsoon climate. After sampling, any visible gravel, roots and other plant materials were discarded and then the soil sample was sieved (<2 mm). The pH of the soil was 7.19, 20.13 g⋅kg^–1^ soil organic matter (SOM), 1.71 g⋅kg^–1^ total nitrogen (TN), 68.5 mg⋅kg^–1^ available nitrogen (AN), available phosphorus (AP) 13.9 mg⋅kg^–1^, 48.2 mg⋅kg^–1^ available potassium (AK). It was used as the basic cultivation material for subsequent experiments.

Aerobic composting was carried out by mixing chicken manure and rice husk according to C/N of 25 and controlling moisture of about 55%. The seed germination index (GI) was used to determine the degree of maturity of the compost, which was determined according to Yang et al. (2021). Throughout the composting process, GI values were monitored every day and compost products with a GI value of 100% were set aside in time to be used as organic fertilizer products for subsequent studies.

### 2.2 Extraction of DOM from organic fertilizer and separation of its hydrophilic and hydrophobic components

According to the method of Zheng et al. (2022), the DOM was extracted from organic fertilizer. The DOM from organic fertilizer was separated by XAD-8 resin (Leenheer, 1981) and then divided into hydrophilic and hydrophobic components. That is, after the pretreatment of XAD-8 resin, the organic fertilizer DOM was passed through the XAD-8 resin column at a flow rate of 1 ml⋅min^–1^, washed with 1.5 times the column volume of ultrapure water, and the component passing through the resin column was collected, then pH adjusted to 2 with 6 mol⋅L^–1^ hydrochloric acid solution and washed with 1 time the column volume of 0.01 mol⋅L^–1^ HCl solution to obtain the hydrophilic component. The resin column was then backwashed with 0.25 times the column volume of 0.1 mol⋅L^–1^ NaOH solution, followed by 1.5 times the column volume of ultrapure water, and the backwash solution was collected to obtain the hydrophobic component.

### 2.3 Experimental site and treatments

According to the principle of equal nutrients (total N input of 0.2 g⋅kg^–1^ soil), the treatments of chemical fertilizer (treatment CF), chemical fertilizer with organic fertilizer DOM hydrophilic components (treatment H), and chemical fertilizer with organic fertilizer DOM hydrophobic components (treatment P) were applied. The amount of DOM components added in H treatment and P treatment was determined according to the amount of hydrophilic and hydrophobic components extracted and separated from each gram of the soil that corresponding to organic fertilizer input of 7500 kg⋅ha^–1^. After the fertilizer was fully mixed with the test soil (from 2.1), DOM components were added in the form of watering, and the soil moisture was controlled at 70% field capacity. A total of 100 g of soil from each fertilizer treatment was incubated in 250 ml mill-mouth conical flasks with 3 replicates. Samples were taken after 24 days of incubation. Soil samples of 100 g were collected and divided into three portions for subsequent physical and chemical properties as well as biological measurements.

### 2.4 Soil characteristics, determination of extracellular enzyme activity, and calculation of nitrogen conversion rates

Soil carbohydrates were determined using the phenol-sulphuric acid method (Nguyen-Sy et al., 2021) and soil phenolics was determined using the Folin-Ciocalteu method (Morita, 1980). Soil ammonium nitrogen (NH_4_^+^-N) and nitrate nitrogen (NO_3_^–^-N) were determined by flow analyzer (Xiao et al., 2022), and soil microbial biomass nitrogen (MBC, MBN) was determined by chloroform fumigation-extraction method (Brookes et al., 1985). Soil protease activity was determined by the Folin-Ciocalteu colorimetric method (Lowry et al., 1951).

The inorganic N in the soil was calculated according to Eq. (1).


(1) $$I\,n\,o\,r\,g\,a\,n\,i\,c\,N=N\,H_{4}^{+}‒N+N\,O_{3}^{‒}‒N$$

The quantification of nitrified N (%) from the mineralized N was performed by using the following Eq. (2).


(2) NitrifiedN=N⁢O3‒‒NI⁢n⁢o⁢r⁢g⁢a⁢n⁢i⁢c⁢N100*%

Where Inorganic N is the sum of ${\text{NH}}_{4}^{+}$-N (mg⋅kg^–1^) and ${\text{NO}}_{3}^{‒}$-N (mg⋅kg^–1^).

The Nitrogen mineralization rate in the soil was calculated according to Eq. (3).


(3) $$N\,m\,i\,n=\frac{\begin{array}{c} {}[{\left(NH_{4}^{+}‒N\right)}_{\text{f}}+{\left(NO_{3}^{‒}‒N\right)}_{\text{f}}‒\\ {\left(NH_{4}^{+}‒N\right)}_{\text{i}}‒{\left(NO_{3}^{‒}‒N\right)}_{\text{i}}] \end{array}}{T_{\text{d}}}$$

Where i and f indicate mineral N concentrations before and after aerobic incubation, respectively, and Td indicates incubation time in days.

### 2.5 Soil DNA extraction and bacterial community determination

A 0.5 g fresh soil was taken for each sample used for extraction of genomic DNA. The purity and concentration of DNA were determined using a Thermo NanoDrop One. The genomic DNA was used as a template for PCR amplification using specific primers with barcode and TaKaRa Premix Taq^®^ Version 2.0 (TaKaRa Biotechnology Co., Dalian, China) according to the selection of sequencing region. PCR amplification of soil bacterial 16SrRNA: PCR amplification of the 16S V3 -V4 variable region of bacterial 16S rRNA was performed using primers (5′-ACTCCTACGGGGAGGCAGCA-3′) and (5′-GGACTACHVGGGTWTCTAAT-3′), and the PCR instrument was BioRadS1000 (Bio- Rad Laboratory, CA). Cycling conditions were as follows: pre-denaturation (94°C for 5 min) 30 cycles (denaturation: 94°C for 30 s, annealing: 52°C for 30 s, extension: 72°C for 30 s) extension (72°C for 10 min) After pooling in equimolar quantities, each PCR product was mixed (Supplementary Table 1). The mixed PCR products were recovered using E.Z.N.A.^®^ Gel Extraction Kit (Omega, USA) and the target DNA fragments were recovered by elution with TE buffer. Afterward, QuantiFluor™-ST (Promega, USA) was used for assay quantification and mixed as required. Library construction was performed according to the standard procedure of NEBNext^®^ Ultra™ II DNA Library Prep Kit for Illumina^®^ (New EnglandBiolabs, USA). The constructed amplicon libraries were subjected to PE250 sequencing using the Illumina Nova 6000 platform (Guangdong Magigene Biotechnology Co., Ltd. Guangzhou, China).

### 2.6 Statistical and data analysis

One-way analysis of variance (ANOVA) performed using SPSS 27.0 software (IBM SPSS Statistics 27, IBM Corp., Armonk, NY, USA). Operational taxonomic units (OTUs) with relative abundance greater than 0.1% in all treatments were selected to construct microbial networks based on Spearman’s correlation *r* > 0.7, *p* < 0.01 using the “psych” package in R 4.3.0, and the connectivity between the connected nodes of the network were calculated using the “igraph” package, and Gephi software (v 0.9.2) was used to map the symbiotic network and estimate the topological properties of the network. Pearson correlation was used to assess the relationship between soil NH_4_^+^-N content and soil microbial properties under treatment H and P where organic fertilizer DOM components was added. Random forest analysis was also conducted in R 4.3.0 to explore the contribution of soil properties and microbial composition to soil nitrogen retention capacity.

## 3 Results and discussion

### 3.1 Effects of organic fertilizer DOM components on soil properties

**FIGURE 1:**
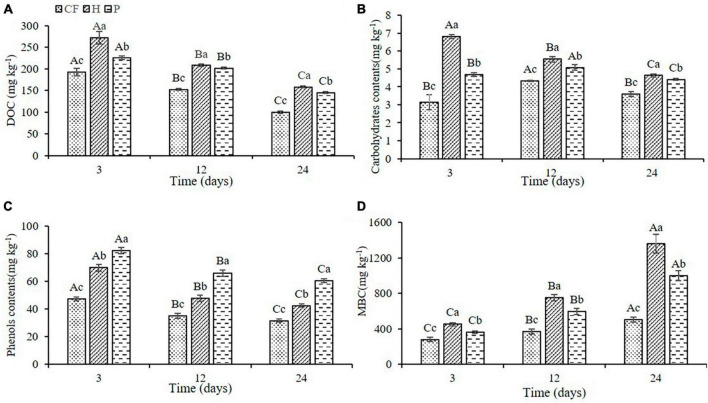
Changes of **(A)** DOC **(B)** carbohydrates **(C)** phenols **(D)** MBC contents in soil after addition of different organic fertilizer DOM components. Different uppercase letters indicated significant differences in different soil incubation stages, and different lowercase letters indicated significant differences in different treatments within the same incubation time, according to one-way ANOVA and LSD multiple comparison test (*P* < 0.05).

Adding the organic fertilizer DOM components significantly increased the content of dissolved organic carbon (DOC) in soil, and the DOC content induced by hydrophilic components was significantly higher than that by hydrophobic components (*H* > *P* > CF, *P* < 0.05; Figure 1A). Furthermore, the DOC content showed a trend of decrease throughout the time of incubation, which was due to the consumption of carbon source by the large proliferation of microorganisms (He et al., 2020). Adding the hydrophilic component of organic fertilizer DOM significantly increased the content of carbohydrates in soil (*H* > *P* > CF, *P* < 0.05; Figure 1B), whereas adding hydrophobic component significantly increased the content of phenols in soil (*P* > *H* > CF, *P* < 0.05; Figure 1C), which was related to the property of organic fertilizer DOM itself. Moreover, the microbial biomass carbon (MBC) content of the soil was significant increased throughout the culture process, which was in contrast to the previous decrease in the content of the various carbon-containing compounds, suggesting that microbe assimilated a large amount of carbon sources into the microbiomass. It is worth noting that DOM hydrophilic components caused a significantly higher MBC content compared with hydrophobic components (Figure 1D), which may be related to the efficiency of microbial carbon source use (Zheng et al., 2021), while higher carbon availability is more conducive to microbial N assimilation (Wang et al., 2019). Therefore, it can be assumed that a higher carbohydrate content under the condition of additional hydrophilic components will increase the N utilization efficiency of microorganisms, resulting in an increase in microbial assimilation of soil nitrogen enabling more nitrogen to be preserved in microbes in the form of microbial biomass nitrogen (MBN), which may alter the characteristics of soil nitrogen transformation.

### 3.2 Effects of organic fertilizer DOM components on soil nitrogen transformation properties

**FIGURE 2:**
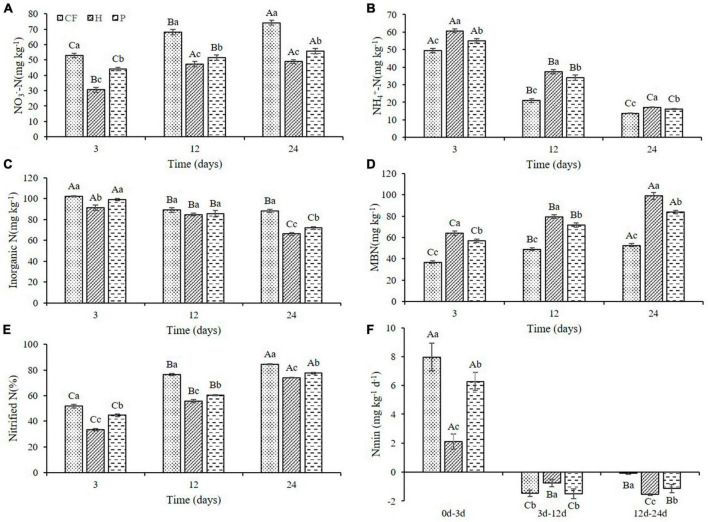
Variations of **(A)** NO_3_^–^-N **(B)** NH_4_^+^-N **(C)** inorganic N **(D)** MBN contents and **(E)** nitrified N **(F)** Nmin in soil after addition of different organic fertilizer DOM components. Different uppercase letters indicated significant differences in different soil incubation stages, and different lowercase letters indicated significant differences in different treatments within the same incubation time, according to one-way ANOVA and LSD multiple comparison test (*P* < 0.05).

To further investigate whether the changes in nitrogen supply characteristics were caused by the differences in carbon availability in DOM hydrophilic and hydrophobic treatments, and if more nitrogen is conserved in the microbiomass, the mineral nitrogen contents and nitrogen transformation characteristics in soil were analyzed. The NO_3_^–^-N content in all soil samples increased with the increase of culture time, while the NH_4_^+^-N content decreased (Figures 2A, B), which was attributed to the nitrification during the culture process. It is worth noting that soil NO_3_^–^-N content under the treatments of adding DOM components (treatment H and P) is significantly lower than that of CF treatment (Figure 2A). Soil NH_4_^+^-N content was the highest in treatment H and the lowest in treatment CF at the same cultivation time (Figure 2B). Moreover, additional organic fertilizer DOM fractions in soil slowed down the net soil nitrification rate (Figure 2E), as the soil notified N was the highest in treatment CF and the lowest in treatment H throughout the culture process. This is consistent with the results of Huang et al. (2022), yet, adding the components of organic fertilizer DOM increased soil NH_4_^+^-N content compared to CF treatment in this study. Combined with NH_4_^+^-N content, nitrification slowdown is likely to be one of the reasons why the total content of NH_4_^+^-N is *H* > *P* > CF. Soil microbial biomass nitrogen (MBN) plays a key role in maintaining soil fertility and is considered to be a biologically active nitrogen reservoir in soil (Hong et al., 2015). Soil MBN content in this study showed an increasing trend in all samples throughout the culture process and always exhibited as the highest in treatment H and the lowest in treatment CF at the same culture time (Figure 2D). This indicated that organic fertilizer DOM components significantly increased soil microbial biomass. The increase in MBN also reflected the increase in soil N storage capacity and the improvement of soil N sustained supply capacity (Chun-mei et al., 2023). In addition, adding the organic fertilizer DOM components decreased soil inorganic N content and N mineralization rate (Nmin < 0) (Figures 2C, F), suggesting that organic fertilizer DOM enhances microbial assimilation of nitrogen, which corroborated with the increase in MBN content (Figure 2D). On the other hand, adding the organic fertilizer DOM components significantly increased the activity of soil protease (Supplementary Figure 1), which could promote the mineralization of organic nitrogen to NH_4_^+^-N (Greenfield et al., 2021), and this is most likely another reason for the increase in NH_4_^+^-N content between treatments. In summary, the increased content of NH_4_^+^-N in the treatment with additional organic fertilizer DOM components could be resulted from the slowed down nitrification rate which in return preserves NH_4_^+^-N. At the same time, the fixation of inorganic nitrogen by microorganisms and the mineralization of organic nitrogen were increased, and addition of the hydrophilic components of DOM of organic fertilizer intensified this process.

### 3.3 Relationship between soil NH_4_^+^-N content, net nitrification rate and soil properties and their contribution to MBN and NH_4_^+^-N content

**FIGURE 3:**
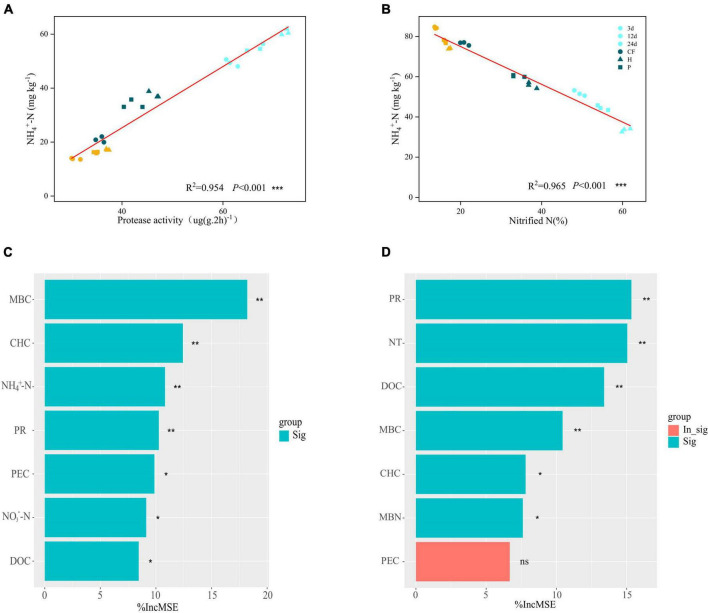
The relationship between NH_4_^+^-N content and **(A)** protease activity and **(B)** nitride N after addition of different organic fertilizer DOM components. Random forest analysis, contribution of soil environmental factors to **(C)** MBN contents **(D)** NH_4_^+^-N contents. Asterisks show that different factors contribute significantly to MBN or NH_4_^+^-N (***P < 0.001, **P < 0.01, *P < 0.05, ^ns^*P* > 0.05).

Soil NH_4_^+^-N content was significantly and negatively correlated with net nitrification rate (Nitride N) (*R*^2^= 0.965, *P* < 0.01) and positively correlated with protease activity (*R*^2^= 0.954, *P* < 0.01) (Figures 3A, B). Adding the organic fertilizer DOM components decreased the soil net nitrification rate (Figure 2E) and increased the protease activity (Supplementary Figure 1) throughout the incubation stage, which further determined that the addition of organic fertilizer DOM components increased the soil NH_4_^+^-N content due to the slowed down nitrification and the increase of organic nitrogen mineralization capacity. The contribution of soil properties to MBN and NH_4_^+^-N contents was further explored by random forest analysis (Figures 3C, D). The results showed that MBC was the most important factor influencing the MBN content and the contribution of carbohydrates was greater than that of phenolics (Figure 3C), which suggests that the higher MBN content induced by organic fertilizer DOM hydrophilic components was due to the higher bioavailability of carbon. The availability of organic carbon components affects microbial nitrogen assimilation, which is an energy-consuming processes. Higher carbon availability is more favorable for microbial N assimilation (Wang et al., 2019). In addition, the contribution of NH_4_^+^-N content was greater than NO_3_^–^-N, indicating that the addition of DOM components of organic fertilizer increased the assimilation of NH_4_^+^-N by soil microbiomass, which enabled nitrogen to be preserved in the microbial mass. On the other hand, protease activity (PR) and nitrification rate (NT) were the main factors affecting NH_4_^+^-N content, and carbohydrate content also contributed significantly to NH_4_^+^-N content, while phenolic content did not (Figure 3D). It is again confirmed that the hydrophilic components of organic fertilizer DOM caused increase of protease activity promoted the mineralization of organic nitrogen and slowed down nitrification, which resulted in the preservation of NH_4_^+^-N in soil.

### 3.4 Effects of organic fertilizer DOM components addition on soil bacterial community composition

Table 1 summarizes the alpha diversity indices of the samples. According to shannon index, at the end of incubation, CF treatment had the lowest community diversity among all samples, while H treatment had the highest. In addition, the microbial diversity of H treatment was significantly higher than that of P treatment with the increase of incubation time (*P* < 0.05). These results indicate that soil microorganisms responded differently to different components of organic fertilizer DOM, and hydrophilic components significantly increased soil microbial diversity.

**TABLE 1:** Effects of different DOM components from organic fertilizer on the microbial diversity index.

Time	Treatments	Richness	Shannon
	CF	4232.67 ± 78.02 Aa	9.34 ± 0.03 Ba
3 days	H	4114.67 ± 54.92 Ba	9.20 ± 0.03 Cb
	P	4173.67 ± 33.47 Aa	9.33 ± 0.06 Ba
	CF	4175.67 ± 40.65 Ab	9.32 ± 0.04 Bb
12 days	H	4306.67 ± 48.01 ABa	9.41 ± 0.05 Ba
	P	4296.00 ± 83.86 Aab	9.41 ± 0.04 ABa
	CF	4295.33 ± 43.02 Aa	9.40 ± 0.03 Ac
24 days	H	4394.00 ± 143.85 Aa	9.55 ± 0.01 Aa
	P	4211.33 ± 137.91 Aa	9.46 ± 0.04 Ab

Different uppercase letters indicated significant differences in different soil culture stages, and different lowercase letters indicated significant differences in different treatments within the same culture time, according to one-way ANOVA and LSD multiple comparison test (*P* < 0.05).

**FIGURE 4:**
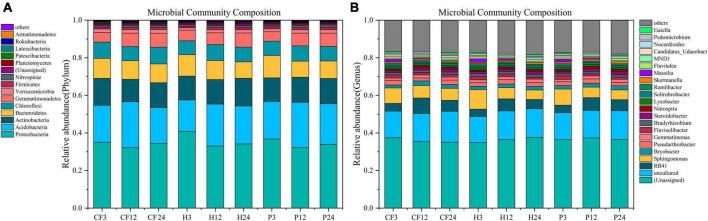
The composition of bacterial [**(A)** phylum level **(B)** genus level] communities after addition of different organic fertilizer DOM components.

The seven phylum with the highest average relative abundance in all samples were *Proteobacteria*, *Acidobacteria*, *Actinobacteria*, *Bacteroidetes*, *Chloroflexi*, *Gemmatimonadetes*, and *Verrucomicrobia*. *Proteobacteria* was the dominant phylum in all samples (Figure 4A). Species identified at the genus level were dominated by *Sphingomonas* and *RB41* with mean relative abundance of 6.005 and 5.447%, respectively, and followed by *Pseudarthrobacter*, *Flavisolibacter*, *Bryobacter*, *Bradyrhizobium*, *Gemmatimonas*, *Lysobacter*, *Ramlibacter*, *Flavitalea*, *Steroidobacter*, *Nitrospira*, *Solirubrobacter*, *Candidatus_Udaeobacter*, *Skermanella*, *Massilia*, *MND1* (Figure 4B). Among them, *Pseudarthrobacter* and *Bradyrhizobium* carry abundance of nitrogen-fixing genes (Tang et al., 2022; Miao et al., 2023), while *Nitrospira* is an iconic ammonia-oxidizing bacteria that oxidizes N_2_O to nitrate and nitrite (Daims et al., 2015). *Sphingomonas* can convert nitrite nitrogen (NO_2_^–^-N) to NO_3_^–^-N in the nitrification process, which is crucial in the nitrification process (Gou et al., 2023). The abundance of *Sphingomonas* decreased throughout the culture process (Figure 4B), which corroborates with the weakening of nitrification. *Gemmatimonas* is associated with nitrogen metabolism (Yu et al., 2022), which promotes the mineralization of soil organic nitrogen. Although it is not clear how the specific mechanism of their metabolism works, it is still worth noting that the abundance of *Gemmatimonas* significantly negative correlated with the NH_4_^+^-N content of the soil in this study (Supplementary Figure 2). *Gaiella* can assimilate carbohydrates, organic acids and amino acids, and also reduce the conversion of NO_3_^–^ to NO_2_^–^ (Lv et al., 2022). Whereas, adding organic fertilizer DOM hydrophilic component significantly increased the relative abundance of *Gemmatimonas* and *Gaiella* (Figure 4B). It indicated that the hydrophilic organic fertilizer DOM components induced increase in microbial abundance of these two genera increased the metabolic assimilation of NH_4_^+^-N, and led to a decrease in NH_4_^+^-N content in treatment H. In conclusion, the addition of DOM hydrophilic component to the organic fertilizer increased the soil microbial biomass and the relative abundance of *Gemmatimonas* and *Gaiella*, which facilitated the metabolic assimilation of microorganisms to N, resulting in more nitrogen being conserved in the MBN.

### 3.5 Microbiological mechanisms of changes in nitrogen transformation characteristics in soil after addition of organic fertilizer DOM components

**FIGURE 5:**
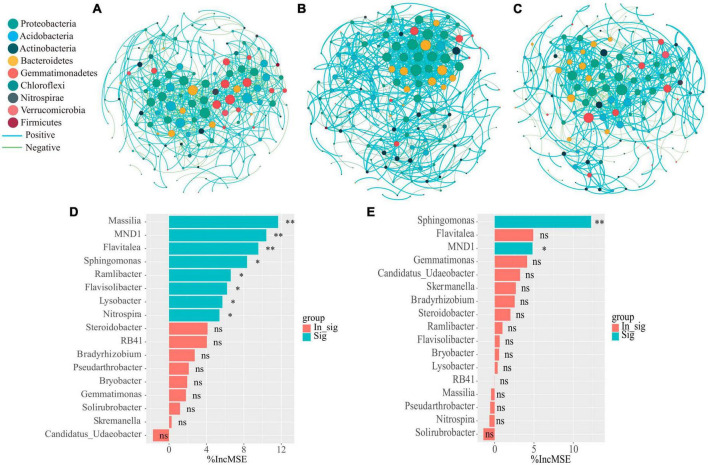
Network co-occurrence analysis of microbial communities of the CF **(A)** and organic fertilizer DOM treatments H **(B)** and P **(C)** samples. Each node represents taxa affiliated at phylum level (based on 16 S rRNA), and the nodes were marked using different color based on the phylum in the network. The size of the node represents the degree of connectivity, and the curve represents the correlation between microorganisms, with blue representing positive correlation and green representing negative correlation. Spearman’s correlation analysis was conducted on bacterial with average relative abundances higher than 0.1% in treatment by R. Then, the correlation with a correlation coefficient (r) greater than 0.7 or less than −0.7 and significance (*P*) less than 0.01 was used to establish microbial networks, which were used to evaluate the relationships among microbial both in treatment. Random forest analysis as a means of determining the effect of bacterial community composition on **(D)** NH_4_^+^-N content as well as **(E)** MBN content in soil. Asterisks show that different relative abundance of microorganisms contribute significantly to MBN or NH_4_^+^-N (**P < 0.01, *P < 0.05, ^ns^*P* > 0.05).

**FIGURE 6:**
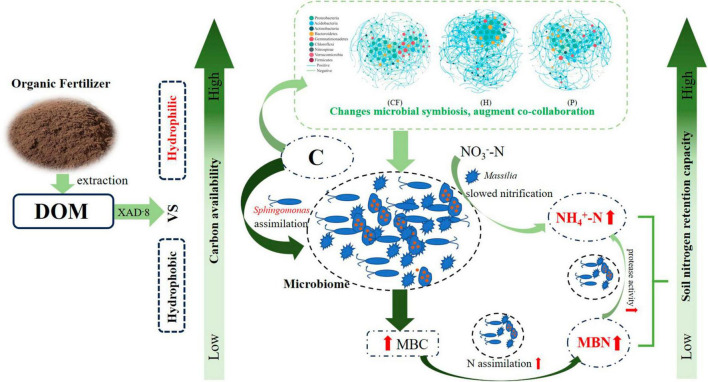
Schematic diagram of DOM components of organic fertilizer increased soil nitrogen retention capacity.

In order to clarify the effect of organic fertilizer DOM components addition on the structure of key soil bacterial communities, the microbial co-occurrence network was mapped (Figure 5). Its network characteristics can represent the response of microbial communities to environmental changes (Lindstrom et al., 2019), while the edges of the network indicate the interactions between microorganisms, and the degree of modularity indicates the diversity of microbial modules. In this study, it was found that the number of organic fertilizer DOM hydrophilic components (treatment H) added higher edges than treatment CF and P, indicating an increased interactions between microorganisms in treatment H. In addition, the modularity of both H and P treatments was lower than that of CF treatment and the positive correlation between microorganisms was also higher in both treatments (Figure 5 and Supplementary Table 2). A community where most of the microbial members are connected through positive correlation is considered unstable; members of these communities respond to environmental changes, resulting in positive feedback and co-collaboration (Wang C. et al., 2023). The results showed that the addition of DOM components of organic fertilizer made the microbial symbiotic network more diverse and the synergistic effect between microorganisms stronger, which was just in line with the results of the diversity index (Table 1), and was of great significance for the assimilation and retention of nitrogen by microorganisms. The results showed that the addition of organic fertilizer DOM components resulted in a more diverse microbial symbiotic network and greater synergies between microorganisms. The addition of organic fertilizer DOM hydrophilic components increased the synergistic interactions between microorganisms and the microbial network complexity, but the addition of organic fertilizer DOM hydrophobic components decreased the microbial interactions. Adding the hydrophilic components of organic fertilizer DOM enhanced microbial interactions and synergism, and increased soil nitrogen fixation, allowing nitrogen to be retained in the microbiota. In all networks, the addition of hydrophilic components of organic fertilizer DOM increased the number of key nodes (with high connectivity), and most of the key nodes belonged to *Proteobacteria* (Supplementary Table 3), which is a key functional microbial group in soil and plays a crucial role in soil carbon, nitrogen and sulfur cycling. In addition, *Proteobacteria* also can participate in the degradation of carbon-containing compounds (Spain et al., 2009). It is noteworthy that *Sphingomonas* appeared at key nodes under all treatments of organic fertilizer DOM components, which may be related to the degradation of macromolecular compounds (Song et al., 2021). The contribution of bacterial communities to the effect of NH_4_^+^-N and MBN content in soil was further explored by random forest analysis (Figures 5D, E). The results showed that *Massilia* was the most important species affecting NH_4_^+^-N content, followed by *MND1* and *Flavitalea*. *Sphingomonas* was the most important species affecting MBN content in soil and *Massilia* showed a negative contribution although the effect was insignificant. *Massilia* is involved in the degradation of aromatic compounds and can metabolize ammonia, nitrate and varies amino acids and other substances (Holochova et al., 2020). *Flavitalea* also plays an important role in the nitrogen cycle and the addition of hydrophilic components of organic fertilizer DOM significantly increased its abundance (Figure 4B). The modular analysis of the nodes to select the species with the highest degree of connectivity (greater than or equal to 25) (Supplementary Table 3) showed that the addition of hydrophilic component of organic fertilizer DOM increased the number of key nodes in the soil, and the key nodes were mainly *Sphingomonas* and *Massilia*. Combined with the results of random forest analysis, it can be concluded that the changes in the abundance of *Sphingomonas* and *Massilia* contributed the most to the changes in soil microbial community composition, soil NH_4_^+^-N and MBN content after hydrophilic components of organic fertilizer DOM was added. Based on the previous discussion, the addition of hydrophilic components of organic fertilizer DOM changed the microbial process of soil nitrogen transformation, thereby increased the soil nitrogen retention ability, and the specific process is shown in Figure 6.

## 4 Conclusion

This study showed that the addition of organic fertilizer DOM components of significantly increased the contents of MBN and NH_4_^+^-N in soil, and thus increased the nitrogen retention capacity of soil. Compared with the addition of hydrophobic components, the hydrophilic components of organic fertilizer DOM has a higher biological availability of carbon, the process of nitrogen retention capacity in soil was strengthened by hydrophilic components of DOM. The relationship between microorganisms become more interconnected after adding hydrophilic components of DOM, *Sphingomonas* and *Massilia* were key species affecting nitrogen retention capacity and nitrogen supply characteristics of soil.

## Data availability statement

The original contributions presented in the study are included in the article/Supplementary material, further inquiries can be directed to the corresponding author.

## Author contributions

YW: Conceptualization, Data curation, Funding acquisition, Methodology, Writing – review and editing. YG: Conceptualization, Data curation, Methodology, Writing – original draft. YD: Methodology, Validation, Writing – original draft. XX: Methodology, Validation, Writing – original draft. YZ: Data curation, Visualization, Writing – original draft. LL: Methodology, Visualization, Writing – original draft. ZX: Conceptualization, Formal analysis, Funding acquisition, Validation, Writing – review and editing.
