# Supplementary material for: DOM hydrophilic components of organic fertilizers increased the soil nitrogen retention capacity and succession of the microbial community
Source: Front Microbiol. 2023 Dec 6;14:1320302. doi: 10.3389/fmicb.2023.1320302 (PMC10730659; doi:10.3389/fmicb.2023.1320302)
Supplement: Supplementary file 1 [file Data_Sheet_1.docx]

Supplementary Material

**TABLE S1** PCR reaction system

| The amplification procedure | | components | volume |
| --- | --- | --- | --- |
| 95℃ 30s |  | 2xqPCRmix | 5ul |
| 95℃ 10s | 40cycle | F primers (10pmol^.^ul^-1^) | 0.25ul |
| 60/65℃ 30s |  | R primers (10pmol^.^ul^-1^) |  |
| 72℃ 30s |  |  | 0.25ul |
| 95℃ 15s |  | DNA formwork | 2ul |
| 60℃ 60s | One test per 0.3°C of temperature rise | ddH_2_O | 2.5ul |
| 95℃ 15s |  | total | 10ul |


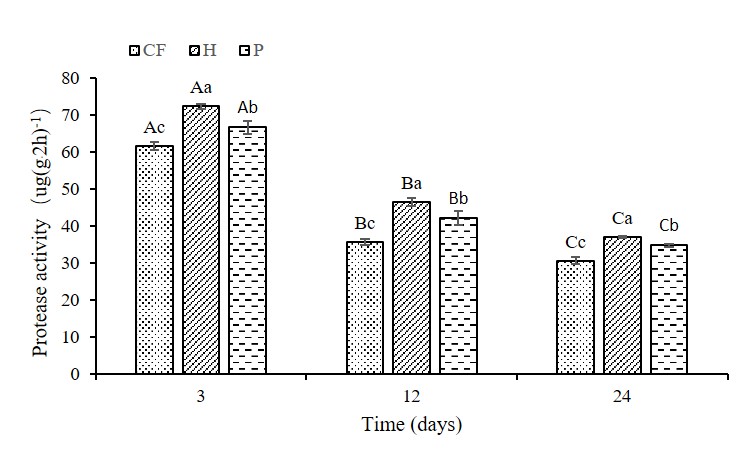


**FIGURE S1** Changes of protease activity after addition of different organic fertilizer DOM components. Different uppercase letters indicated significant differences in different soil incubation stages, and different lowercase letters indicated significant differences in different treatments within the same incubation time, according to one-way ANOVA and LSD multiple comparison test (P<0.05).


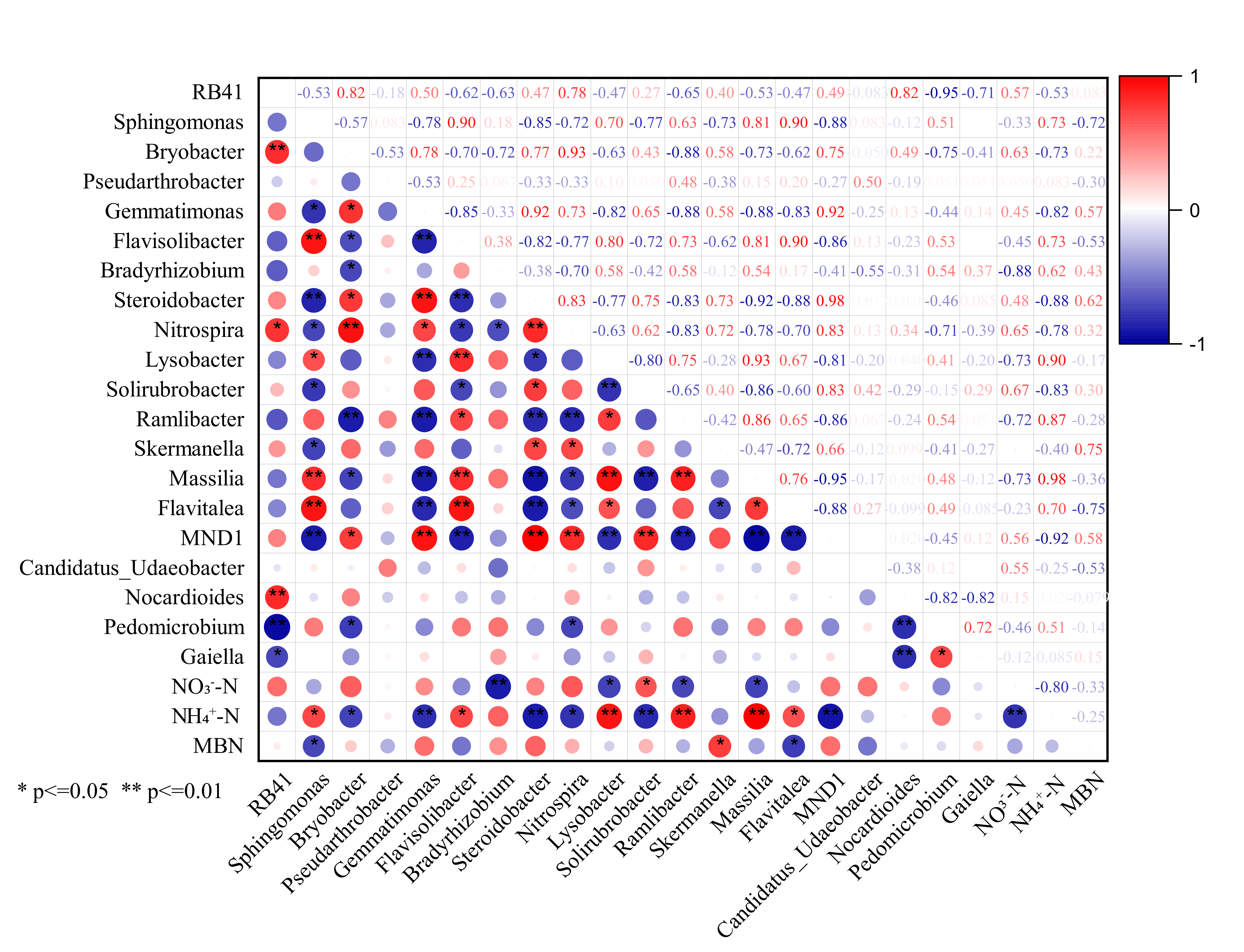


**FIGURE S2** The relationship between bacterial genus level community abundance and soil nitrogen content is shown using Spearman correlation analysis. Red color indicates positive correlation and blue color indicates negative correlation. Significance levels: * P < 0.05, ** P < 0.01.

**TABLE S2** Topological indices of each bacterial networks in FIGURE 5.

|  | Nodes | Edges | Average degree | Network density | Modularity | Average clustering coefﬁcient | Positive correlation (%) | Negative correlation (%) |
| --- | --- | --- | --- | --- | --- | --- | --- | --- |
| CF | 161 | 631 | 7.839 | 0.049 | 2.565 | 0.422 | 58.47 | 41.53 |
| H | 164 | 761 | 9.28 | 0.057 | 1.592 | 0.432 | 65.97 | 34.03 |
| P | 164 | 555 | 6.768 | 0.042 | 1.658 | 0.399 | 65.22 | 34.78 |

**TABLE S3** Key nodes of each different microbial networks in FIGURE 3

| Treatment | Nodes_id | Degree | Phylum | Genus |
| --- | --- | --- | --- | --- |
| CF | OTU_75 | 26 | *Proteobacteria* | *Massilia* |
|  | OTU_101 | 25 | *Bacteroidetes* | *Parasegetibacter* |
|  | OTU_186 | 25 | *Acidobacteria* | *uncultured* |
|  | OTU_3 | 25 | *Acidobacteria* | *RB41* |
| H | OTU_75 | 32 | *Proteobacteria* | *Massilia* |
|  | OTU_62 | 31 | *Bacteroidetes* | *Hymenobacter* |
|  | OTU_113 | 30 | *Proteobacteria* | *Sphingomonas* |
|  | OTU_14 | 30 | *Proteobacteria* | *Lysobacter* |
|  | OTU_78 | 29 | *Bacteroidetes* | *NA* |
|  | OTU_1 | 28 | *Proteobacteria* | *Sphingomonas* |
|  | OTU_235 | 28 | *Acidobacteria* | *NA* |
|  | OTU_916 | 28 | *Proteobacteria* | *Massilia* |
|  | OTU_42 | 26 | *Proteobacteria* | *Ramlibacter* |
|  | OTU_884 | 26 | *Proteobacteria* | *Sphingomonas* |
|  | OTU_137 | 25 | *Proteobacteria* | *NA* |
|  | OTU_2871 | 25 | *Proteobacteria* | *MND1* |
| P | OTU_1 | 25 | *Proteobacteria* | *Sphingomonas* |
